# A MICROLONGITUDINAL APPROACH TO STUDYING GRANDMOTHER-GRANDCHILD DAILY INTERACTIONS

**DOI:** 10.1093/geroni/igz038.1041

**Published:** 2019-11-08

**Authors:** Shelbie Turner, Shannon T Mejia, Robert S Stawski, Karen Hooker

**Affiliations:** 1 Oregon State University, Corvallis, Oregon, United States; 2 University of Illinois, Urbana-Champaign, Champaign, Illinois, United States

## Abstract

Research suggests that grandparent-grandchild dyads shift in degree of solidarity over extended periods of time (e.g. Moorman & Stokes, 2016), but no work has considered grandparent-grandchild interactions microlongitudinally. This study utilized microlongitudinal data with an emphasis on intraindividual variability to examine the daily processes associated with relational aspects of grandparenting. Using data from 24 grandmothers in the Personal Understandings of Life and Social Experiences (PULSE) project, we explored how grandmother-reported satisfaction with grandchild interactions impacted grandmothers’ same-day positive and negative affect over 100 days. We first justified the need for microlongitudinal analyses by assessing the degree to which there were within-person shifts in interaction satisfaction over time. Intra-class correlations indicated 86% of the variation in interaction satisfaction was within-persons, warranting an intraindividual variability approach. As such, we then employed multi-level models to examine the within-person and between-person effects of interaction satisfaction predicting same day positive and negative affect. At the within-person level, on days when grandmothers reported higher than their average interaction satisfaction, they reported more positive affect (Estimate = 0.09, SE = 0.03, p = 0.009) and lower negative affect overall that day (Estimate = -0.08, SE = 0.02, p = 0.005). At the between-person level, grandmothers who had, on average, higher interaction satisfaction had more positive affect (Estimate = 0.63, SE = 0.09, p<.0001) and lower negative affect on average (Estimate = -0.53, SE = 0.11, p<.0001).

